# A multimodal approach to early detection of anthracycline-induced cardiotoxicity: complementary roles of left ventricular global longitudinal strain, left atrial reservoir strain, and high-sensitivity troponin I

**DOI:** 10.1186/s44348-026-00071-y

**Published:** 2026-04-13

**Authors:** Ahmet Ferhat Kaya, Mehmet Özbek

**Affiliations:** 1 Department of Cardiology, Van Regional Education and Research Hospital, Van, Türkiye; 2 Department of Cardiology, Faculty of Medicine, Dicle University, Diyarbakır, Türkiye

**Keywords:** Anthracyclines, Breast neoplasms, Cardiotoxicity, Global longitudinal strain, Troponin

## Abstract

**Background:**

Anthracycline-based chemotherapy is highly effective in breast cancer treatment but is limited by dose-dependent cardiotoxicity. Early identification of subclinical myocardial injury is crucial to prevent progression to irreversible dysfunction.

**Objectives:**

To evaluate whether a multimodal surveillance strategy integrating left ventricular global longitudinal strain (LVGLS), left atrial reservoir strain (LASr), and high-sensitivity troponin I (hs-TnI) can predict early anthracycline-induced cardiotoxicity.

**Methods:**

This retrospective cohort study included 50 female breast cancer patients (mean age 49.3 ± 8.5 years) treated between January 2022 and December 2024. Echocardiography and biomarkers were assessed at baseline and 1 month after chemotherapy. Cardiotoxicity was defined as a > 10% reduction in LVEF to < 53%.

**Results:**

Cardiotoxicity occurred in 15 patients (30%). LVGLS, LASr, and hs-TnI significantly changed (all P < 0.001). Independent predictors were LVGLS (aOR 1.33), LASr (aOR 0.77), and hs-TnI (aOR 1.07). hs-TnI showed the highest discriminative ability (AUC 0.940).

**Conclusions:**

LVGLS, LASr, and hs-TnI provide complementary information for early detection of cardiotoxicity.

**Graphical Abstract:**

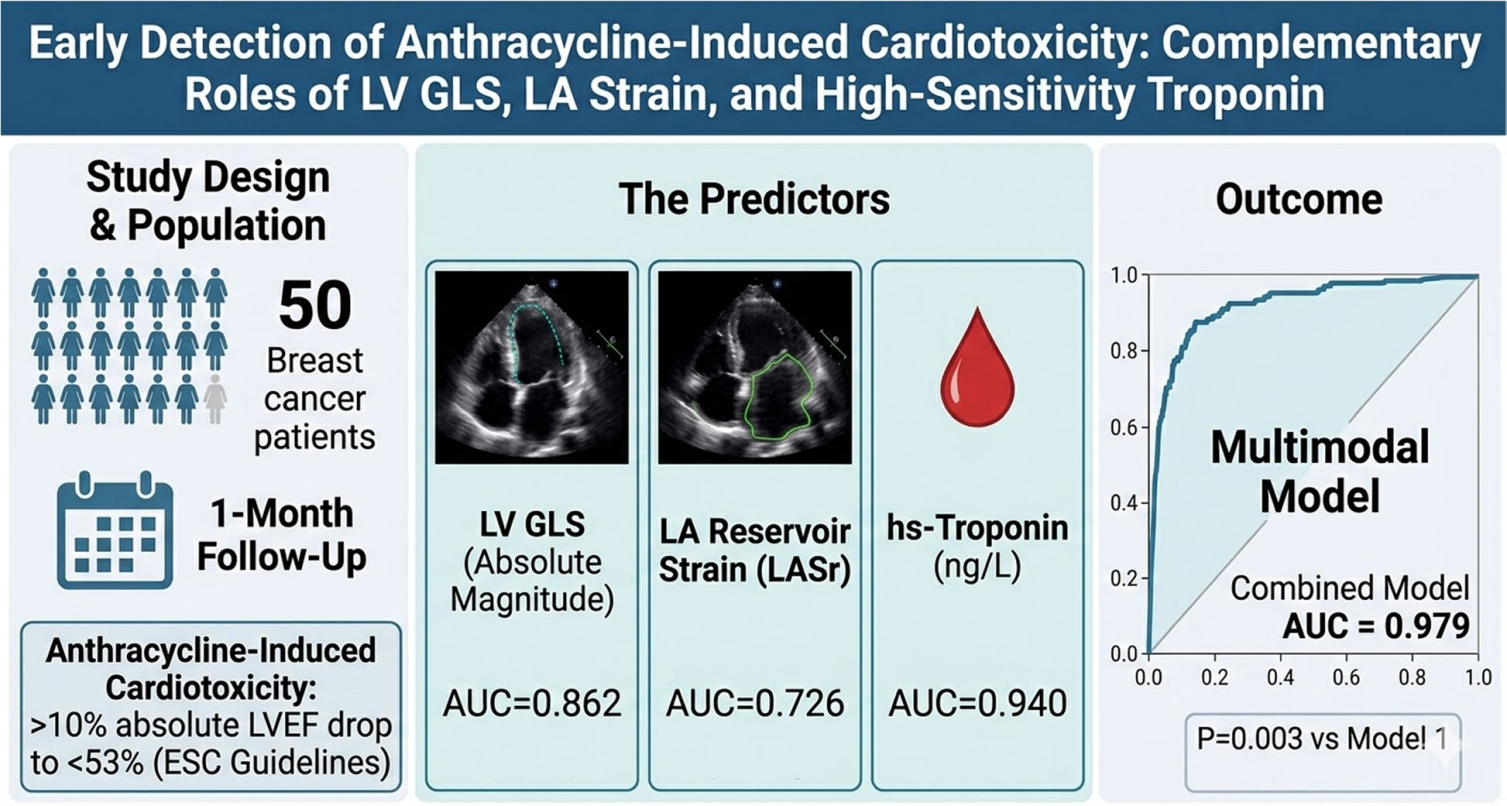

## Introduction

Anthracycline-based chemotherapy remains a cornerstone in the treatment of breast cancer because of its well-established efficacy. However, its clinical use is limited by dose-dependent cardiotoxicity, which may initially manifest as subclinical myocardial dysfunction and subsequently progress to heart failure. As cardiovascular disease represents a leading non-cancer cause of morbidity and mortality among breast cancer survivors, early detection of chemotherapy-related cardiac injury has become a central objective in cardio-oncology practice [1–3].

Transthoracic echocardiography (TTE) is the primary imaging modality for cardiac surveillance in patients undergoing potentially cardiotoxic therapies. Although left ventricular ejection fraction (LVEF) has traditionally been used to define chemotherapy-related cardiac dysfunction, it lacks sensitivity for detecting early myocardial injury. Two-dimensional speckle-tracking echocardiography enables the quantitative assessment of myocardial deformation, and left ventricular global longitudinal strain (LVGLS) has emerged as a sensitive marker of early systolic impairment preceding LVEF decline [4–6].

Beyond ventricular systolic mechanics, atrial function reflects ventricular filling pressure, myocardial compliance, and early diastolic alterations. Left atrial strain analysis, particularly during the reservoir phase (left atrial reservoir strain, LASr), has gained interest as a potential indicator of subtle changes in ventricular–atrial coupling. However, data regarding its role in the early detection of anthracycline-induced cardiotoxicity remain limited [7, 8].

In parallel, cardiac biomarkers such as high-sensitivity troponin I (hs-TnI) provide biochemical evidence of myocardial injury and may identify cardiomyocyte damage before functional impairment becomes clinically evident [9, 10]. Whether the integration of deformation imaging and biochemical markers provides incremental value over single-parameter assessment remains unclear.

Accordingly, this study aimed to evaluate whether a multimodal strategy combining LVGLS, LASr, and hs-TnI improves the early discrimination of anthracycline-induced cardiotoxicity in patients with breast cancer.

## Methods

### Ethics statement

This study was approved by the Dicle University Clinical Research Ethics Committee (No. 18.12.2024/06). Informed consent was waived due to the retrospective design and the use of de-identified patient data extracted from electronic health records. No direct patient contact or intervention occurred, and all data were anonymized to ensure confidentiality. The study was conducted in accordance with the principles of the Declaration of Helsinki.

### Study population

This retrospective study included consecutive patients with breast cancer treated with anthracycline-based chemotherapy between January 2022 and December 2024. Patients aged ≥ 18 years who underwent transthoracic echocardiography (TTE) before treatment initiation and at 1-month follow-up were included.

Exclusion criteria were prior heart failure, significant valvular disease, known coronary artery disease, chronic renal or hepatic failure, active infection, baseline left bundle branch block, and autoimmune disease.

### Echocardiographic assessment

All TTE examinations were performed using standardized imaging protocols in accordance with contemporary European Society of Cardiology (ESC) cardio-oncology recommendations. Conventional echocardiographic parameters were obtained, and two-dimensional (2D) speckle-tracking echocardiography was used to derive left ventricular global longitudinal strain (LVGLS) and left atrial strain parameters from apical four- and two-chamber views.

Left atrial reservoir strain (LASr) was measured during ventricular systole, representing atrial filling. Strain values were averaged over three cardiac cycles. For receiver operating characteristic (ROC) and regression analyses, LVGLS was analyzed as an absolute magnitude to facilitate interpretability.

### Reproducibility analysis

To assess reproducibility, intraobserver and inter-observer variability were evaluated in a random subset of 20 patients. For intraobserver variability, the primary observer repeated the analysis 2 weeks apart, blinded to prior measurements. For inter-observer variability, a second independent observer, blinded to all clinical data and previous measurements, performed the analysis.

Reproducibility was assessed using the intraclass correlation coefficient (ICC) with a two-way mixed-effects model for absolute agreement. The intraobserver ICC for LVGLS was 0.96 (95% CI, 0.91–0.98) and for LASr was 0.93 (95% CI, 0.87–0.97). The inter-observer ICC for LVGLS was 0.92 (95% CI, 0.85–0.96) and for LASr was 0.89 (95% CI, 0.80–0.94), indicating excellent reproducibility.

### Biomarker analysis

Venous blood samples were obtained at baseline and at 1-month follow-up. High-sensitivity troponin I (hs-TnI) was measured using a commercially available assay (manufacturer information blinded for review), with a defined analytical range and limit of detection. The 99th percentile upper reference limit was applied according to assay specifications. hs-TnI was analyzed as a continuous variable (ng/L).

N-terminal pro–brain natriuretic peptide (NT-proBNP) and high-sensitivity C-reactive protein (hs-CRP) levels were analyzed descriptively but were not included in the final prediction models.

### Definition of cardiotoxicity

Cardiotoxicity was defined as a relative decline in LVEF of > 10% to an absolute value of < 53% at the 1-month follow-up echocardiogram compared with baseline. This definition reflects early cardiotoxicity; confirmation of persistent dysfunction requires longitudinal follow-up according to ESC guidelines.

The initiation of cardioprotective therapy (e.g., angiotensin-converting enzyme inhibitors or beta-blockers) was at the discretion of the treating physician and was not standardized.

### Statistical analysis

Continuous variables are expressed as mean ± standard deviation or median (interquartile range), as appropriate. Normality was assessed using the Shapiro–Wilk test. Paired-samples t-test or Wilcoxon signed-rank test were used for within-subject comparisons.

A two-sided P-value < 0.05 was considered statistically significant. Statistical analyses were performed using IBM SPSS version 25.0 (IBM Corp) and supplementary statistical software for ROC curve comparison and internal validation.

### Regression modeling

Given the limited number of outcome events, multivariable logistic regression modeling was restricted to prespecified clinically relevant variables central to the study hypothesis: 1-month LVGLS, 1-month LASr, and 1-month hs-TnI levels.

LVGLS was analyzed as an absolute magnitude to facilitate interpretability and ensure consistent directionality of the odds ratios. Adjusted odds ratios (aORs) with 95% confidence intervals (CIs) were reported.

For regression and receiver operating characteristic (ROC) analyses, 1-month follow-up values were used to reflect early treatment-related myocardial injury.

Collinearity among predictors was assessed using the variance inflation factor (VIF), with a VIF > 2.5 considered indicative of potential multicollinearity.

### Discrimination and nested model comparison

Discriminatory performance was evaluated using ROC curve analysis with calculation of the area under the curve (AUC) and corresponding 95% CIs.

Nested models were constructed to assess incremental predictive value:

1. Model 1: LVGLS
2. Model 2: LVGLS + LASr
3. Model 3: LVGLS + LASr + hs-TnI

Pairwise comparisons of correlated ROC curves were performed using the DeLong method. Optimal cut-off values were determined using the Youden index.

### Internal validation and model stability

To assess model stability and potential overfitting, internal validation was performed using bootstrap resampling (500 iterations).

The optimism-corrected AUC and calibration slope were obtained by subtracting the mean bootstrap optimism from the apparent model performance.

A two-sided P-value < 0.05 was considered statistically significant. Statistical analyses were performed using IBM SPSS version 25.0 (IBM Corp) and supplementary statistical software for ROC curve comparison and internal validation.

## Results

### Baseline characteristics

A total of 50 female patients with breast cancer were enrolled. Fifteen patients (30.0%) met the predefined criteria for chemotherapy-induced cardiotoxicity at the 1-month follow-up.

Baseline demographic and clinical characteristics stratified by cardiotoxicity status are summarized in Table 1. The mean age was 49.3 ± 8.5 years. Diabetes mellitus and hypertension were present in 16 (32.0%) and 20 patients (40.0%), respectively. Baseline LVEF was preserved in all participants. No significant differences were observed between groups in terms of baseline LVEF, LVGLS, LASr, or hs-TnI levels.

**Table 1:** Baseline characteristics of the cohort stratified by cardiotoxicity

Characteristic	Entire cohort (n = 50)	No cardiotoxicity (n = 35)	Cardiotoxicity (n = 15)
Age (years)	49.3 ± 8.5	49.6 ± 8.4	48.7 ± 9.1
BMI (kg/m^2^)	27.6 ± 3.2	27.9 ± 3.2	27.1 ± 3.4
Diabetes mellitus	16 (32.0)	11 (31.4)	5 (33.3)
Hypertension	20 (40.0)	13 (37.1)	7 (46.7)
Tumor stage			
II	28 (56.0)	21 (60.0)	7 (46.7)
III	19 (38.0)	13 (37.1)	6 (40.0)
IV	3 (6.0)	1 (2.9)	2 (13.3)
Mastectomy	47 (94.0)	34 (97.1)	13 (86.7)
Radiotherapy	48 (96.0)	35 (100.0)	13 (86.7)
Anthracycline type			
ADR	12 (24.0)	9 (25.7)	3 (20.0)
DOXO	30 (60.0)	22 (62.9)	8 (53.3)
EPI	8 (16.0)	4 (11.4)	4 (26.7)
LVEF (%)	60.0 (60.0–60.0)	60.0 (60.0–60.0)	60.0 (60.0–60.0)
LVGLS (%)	− 16.8 (− 19.9 to − 13.7)	− 16.4 (− 19.0 to − 14.1)	− 20.1 (− 21.0 to − 13.4)
LASr (%)	24.5 (18.2 to 32.9)	25.0 (18.0 to 33.5)	22.4 (19.4 to 32.8)
hs-TnI (ng/L)	45.4 (30.5 to 66.5)	49.9 (35.1 to 65.2)	36.7 (22.1 to 56.9)
LVEDD (mm)	40.6 ± 6.0	41.0 ± 6.4	39.6 ± 5.0
LVESD (mm)	26.3 ± 6.8	26.6 ± 6.9	25.5 ± 6.7

Values are presented as mean ± standard deviation, number (%), or median (interquartile range). ADR, doxorubicin; DOXO, doxorubicin; EPI, epirubicin; BMI, body mass index; hs-TnI, high-sensitivity troponin I; LASr, left atrial reservoir strain; LVEDD, left ventricular end-diastolic diameter; LVEF, left ventricular ejection fraction; LVGLS, left ventricular global longitudinal strain; LVESD, left ventricular end-systolic diameter

### Changes in conventional echocardiographic parameters

At 1-month follow-up, a significant deterioration in systolic function was observed (Tables 2 and 3). Median LVEF decreased from 59.6% (interquartile range [IQR], 55.0%–60.0%) to 54.7% (IQR, 48.0%–59.0%; P < 0.001).

**Table 2:** One-month follow-up characteristics stratified by cardiotoxicity

Characteristic	Entire cohort (n = 50)	No cardiotoxicity (n = 35)	Cardiotoxicity (n = 15)
LVEF (%)	55.0 (52.0 to 58.0)	57.0 (55.0 to 58.0)	50.0 (50.0 to 52.0)
LVGLS (%)	− 18.1 (− 23.9 to − 15.3)	− 17.2 (− 20.1 to − 14.8)	− 26.3 (− 28.5 to − 20.5)
LASr (%)	19.8 (12.8 to 24.8)	20.8 (17.2 to 26.3)	12.8 (8.9 to 19.3)
hs-TnI (ng/L)	96.0 (53.0 to 127.2)	83.6 (46.6 to 96.5)	148.0 (121.5 to 253.1)
LVEDD (mm)	44.8 ± 5.4	44.9 ± 5.5	44.6 ± 5.3
LVESD (mm)	27.3 ± 6.5	27.5 ± 6.4	26.9 ± 6.9
TAPSE (mm)	18.0 (14.0 to 22.0)	18.0 (14.0 to 22.0)	17.0 (13.5 to 21.0)

Values are presented as median (interquartile range) or mean ± standard deviation. hs-TnI, high-sensitivity troponin I; LASr, left atrial reservoir strain; LVEDD, left ventricular end-diastolic diameter; LVEF, left ventricular ejection fraction; LVGLS, left ventricular global longitudinal strain; LVESD, left ventricular end-systolic diameter; TAPSE, tricuspid annular plane systolic excursion

**Table 3:** Changes in key echocardiographic and biomarker parameters after chemotherapy

Variable	Before chemotherapy (n = 50)	At 1-month follow-up (n = 50)	P-value
LVEF (%)	59.6 (55.0 to 60.0)	54.7 (48.0 to 59.0)	< 0.001
LVEDD (mm)	40.6 ± 5.9	44.8 ± 5.3	< 0.001
LVESD (mm)	26.3 ± 6.8	32.0 ± 6.4	< 0.001
Maximum PA velocity (cm/s)	0.97 (0.50 to 1.49)	0.95 (0.47 to 1.70)	0.328
PA VTI (cm)	20.8 ± 4.0	18.1 ± 5.4	< 0.001
LVOT VTI (cm)	22.7 (12.4 to 34.8)	18.7 (9.0 to 29.0)	< 0.001
Maximum AV velocity (cm/s)	1.6 (0.8 to 1.4)	1.3 (0.8 to 2.1)	0.029
Mitral E (cm/s)	92.4 (41.0 to 200.0)	73.9 (35.0 to 230.0)	< 0.001
Mitral A (cm/s)	92.7 ± 25.7	88.3 ± 20.6	< 0.001
DT (ms)	147.7 (67.0 to 320.0)	148.5 (85.0 to 330.0)	0.571
E/E′ ratio	11.4 (4.1 to 73.0)	9.3 (3.6 to 46.0)	0.003
TAPSE (mm)	21 (11 to 40)	18.4 (9 to 37)	0.003
MPI (Tei index)	0.74 (0.40 to 1.11)	0.79 (0.31 to 1.89)	0.725
RA area (cm^2^)	15.1 (8.6 to 29.0)	13.5 (6.0 to 36.3)	0.001
LAVI (mL/m^2^)	19.1 (9.8 to 34.7)	19.5 (11.6 to 30.6)	0.604
LVGLS (%)	− 17.0 (− 29.2 to − 10.1)	− 19.6 (− 33.8 to − 10.9)	< 0.001
LASr (%)	24.5 (18.2 to 32.9)	19.8 (12.8 to 24.8)	< 0.001
RVGLS (%)	− 15.9 (− 23.5 to − 5.5)	− 17.2 (− 23.3 to − 9.1)	0.016
hs-CRP (mg/L)	2.6 (0.4 to 119.0)	23.8 (0.6 to 126.0)	0.523
NT-proBNP (pg/mL)	97 (16 to 350)	104 (37 to 420)	0.301

Values are presented as median (interquartile range) or mean ± standard deviation. Paired-samples t-test or Wilcoxon signed-rank test was used for within-subject comparisons. AV, aortic valve; DT, deceleration time; hs-CRP, high-sensitivity C-reactive protein; LASr, left atrial reservoir strain; LAVI, left atrial volume index; LVEDD, left ventricular end-diastolic diameter; LVEF, left ventricular ejection fraction; LVESD, left ventricular end-systolic diameter; LVGLS, left ventricular global longitudinal strain; LVOT, left ventricular outflow tract; MPI, myocardial performance index; NT-proBNP, N-terminal pro–brain natriuretic peptide; PA, pulmonary artery; RA, right atrium; RVGLS, right ventricular global longitudinal strain; TAPSE, tricuspid annular plane systolic excursion; VTI, velocity–time integral

Significant increases in left ventricular end-diastolic diameter (LVEDD) and left ventricular end-systolic diameter (LVESD) were observed (both P < 0.001), consistent with early ventricular remodeling. Tricuspid annular plane systolic excursion (TAPSE) decreased significantly (P = 0.003), suggesting early right ventricular involvement.

### Myocardial deformation and biomarker changes

LVGLS significantly worsened after chemotherapy (P < 0.001). In the cardiotoxicity group, LVGLS worsened from − 19.6% at baseline to − 17.0% at 1 month (P < 0.001), indicating impaired myocardial deformation. For regression and ROC analyses, LVGLS was analyzed as an absolute magnitude, such that higher values indicated greater impairment.

LASr significantly decreased at follow-up (P < 0.001), reflecting impaired atrial reservoir function. Right ventricular global longitudinal strain (RVGLS) also showed a modest but significant worsening (P = 0.016).

hs-TnI levels increased significantly from baseline to 1 month (P < 0.001). NT-proBNP and hs-CRP did not demonstrate independent predictive value.

**Fig. 1:**
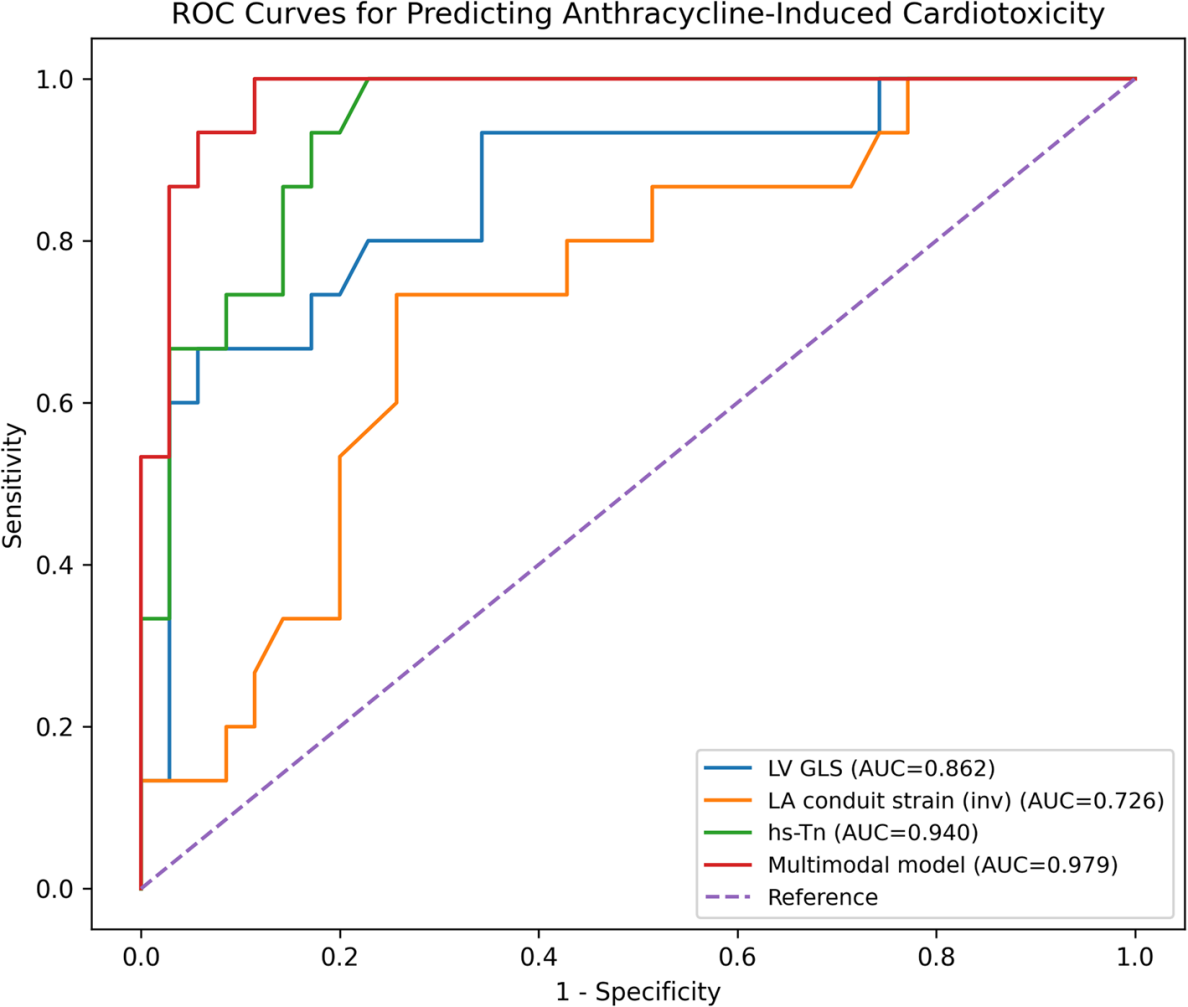
Representative examples of left ventricular global longitudinal strain (LVGLS) and left atrial reservoir strain (LASr) at baseline and 1-month follow-up in patients with and without cardiotoxicity. LVEF, left ventricular ejection fraction

Representative strain examples are shown in Fig. 1.

In a post hoc exploratory analysis, neither RVGLS nor TAPSE remained independent predictors in a multivariable model including LVGLS, LASr, and hs-TnI.

### Multivariable logistic regression analysis

Multivariable analysis identified three independent predictors of cardiotoxicity (Table 4):

1. LVGLS (absolute magnitude): adjusted OR 1.33 (95% CI, 1.01–1.74; P = 0.043)
2. LASr: adjusted OR 0.77 (95% CI, 0.60–0.99; P = 0.038)
3. hs-TnI: adjusted OR 1.07 (95% CI, 1.00–1.13; P = 0.039)

**Table 4:** Independent predictors of cardiotoxicity and ROC performance

Predictor	aOR (95% CI)	P-value	AUC (95% CI)	Cutoff	Sensitivity (%)	Specificity (%)
LVGLS (%)	1.33 (1.01–1.74)	0.043	0.862 (0.745–0.979)	20.2	80.0	77.1
LASr (%)	0.77 (0.60–0.99)	0.038	0.726 (0.574–0.878)	18.5	73.3	68.6
hs-TnI (ng/L)	1.07 (1.00–1.13)	0.039	0.940 (0.879–1.000)	110.7	86.7	85.7

LVGLS values were analyzed as absolute magnitude in regression and ROC analyses. LASr demonstrated an inverse association with cardiotoxicity; therefore, ROC analysis was directionally corrected. aOR, adjusted odds ratio; AUC, area under the curve; CI, confidence interval; hs-TnI, high-sensitivity troponin I; LASr, left atrial reservoir strain; LVGLS, left ventricular global longitudinal strain; ROC, receiver operating characteristic

Each 1% worsening in LVGLS (absolute magnitude) was associated with a 33% increase in cardiotoxicity risk. Lower LASr values were associated with higher risk, indicating an inverse relationship.

**Fig. 2:**
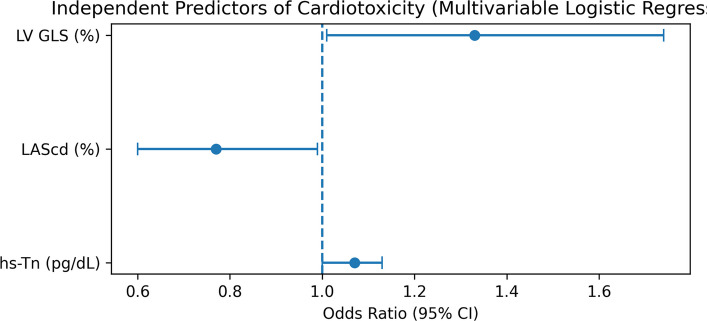
Independent predictors of cardiotoxicity based on multivariable logistic regression analysis. CI, confidence interval; hs-TnI, high-sensitivity troponin I; LASr, left atrial reservoir strain; LVGLS, left ventricular global longitudinal strain

The distribution of adjusted odds ratios is illustrated in Fig. 2.

### Discriminatory performance and nested model comparison

ROC analysis demonstrated the following:

1. LVGLS: AUC 0.862 (95% CI, 0.745–0.979)
2. LASr: AUC 0.726 (95% CI, 0.574–0.878)
3. hs-TnI: AUC 0.940 (95% CI, 0.879–1.000)

Optimal cut-off values are presented in Table 4.

**Fig. 3:**
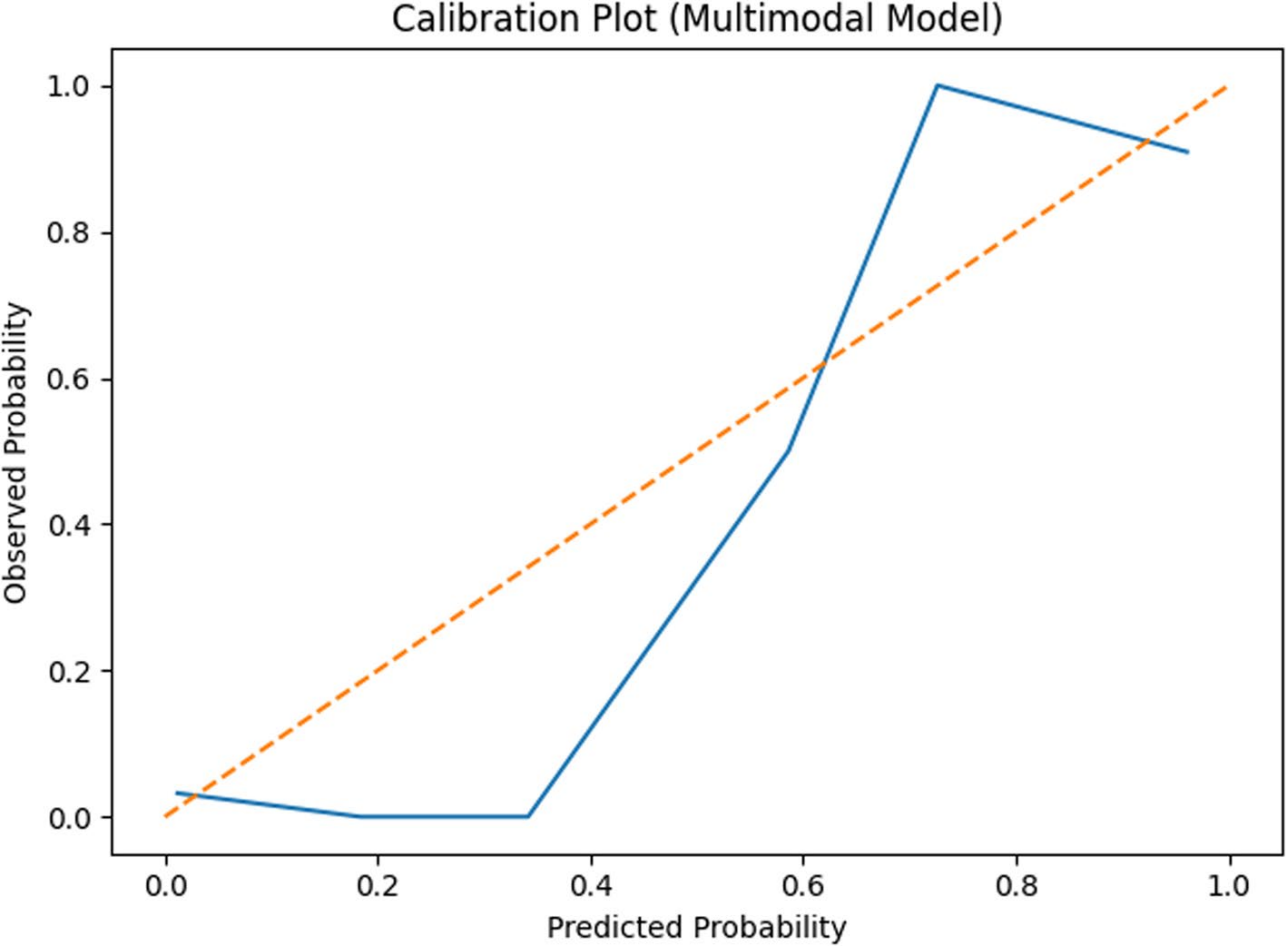
Receiver operating characteristic curves for predicting anthracycline-induced cardiotoxicity. AUC, area under the curve; hs-TnI, high-sensitivity troponin I; LVGLS, left ventricular global longitudinal strain

Nested model analysis showed stepwise improvement in discrimination (Table 5, Fig. 3):

1. Model 1 (LVGLS): AUC 0.862
2. Model 2 (LVGLS + LASr): AUC 0.931 (vs. Model 1, P = 0.017)
3. Model 3 (LVGLS + LASr + hs-TnI): AUC 0.979

**Table 5:** Nested ROC model performance and pairwise AUC comparisons

Model	AUC (95% CI)	Comparison	P-value
Model 1 (LVGLS)	0.862 (0.745–0.979)	Model 1 vs. Model 2	0.017
Model 2 (LVGLS + LASr)	0.931 (0.865–0.996)	Model 1 vs. Model 3	0.003
Model 3 (LVGLS + LASr + hs-TnI)	0.979 (0.954–1.000)	Model 2 vs. Model 3	0.050

Pairwise comparisons between nonadjacent models (Model 1 vs. Model 3) were also significant (P = 0.003). AUC, area under the curve; CI, confidence interval; hs-TnI, high-sensitivity troponin I; LASr, left atrial reservoir strain; LVGLS, left ventricular global longitudinal strain; ROC, receiver operating characteristic

Pairwise comparison using the DeLong method demonstrated a significant improvement between Model 1 and Model 3 (P = 0.003). The addition of hs-TnI to Model 2 showed borderline incremental value (P = 0.050).

### Internal validation and model stability

Variance inflation factor (VIF) analysis demonstrated no evidence of multicollinearity among the predictors (all VIF < 2).

**Fig. 4:**
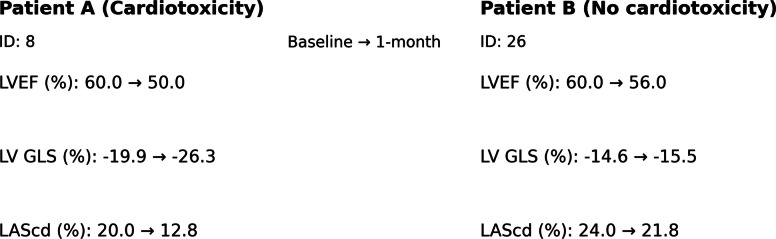
Calibration plot for the multimodal model

Bootstrap internal validation (500 iterations) demonstrated attenuation of the apparent model performance. For the multimodal model (Model 3), the apparent AUC was 0.983 and the optimism-corrected AUC was 0.968. Calibration performance is shown in Fig. 4.

Calibration slope analysis suggested reduced model stability, consistent with potential optimism related to the limited number of outcome events.

## Discussion

In this exploratory pilot study, we evaluated the incremental value of combining left ventricular global longitudinal strain (LVGLS), left atrial reservoir strain (LASr), and high-sensitivity troponin I (hs-TnI) for the early discrimination of anthracycline-induced cardiotoxicity in patients with breast cancer. Our findings suggest that these parameters provide complementary information for early risk stratification. However, the results should be interpreted with caution given the limited sample size and number of outcome events.

### LVGLS and early mechanical alterations

LVGLS demonstrated good discriminatory performance for early cardiotoxicity and remained independently associated with risk in multivariable analysis. These findings are consistent with previous studies showing that longitudinal myocardial deformation is impaired before a measurable decline in LVEF [3, 11–13]. In line with current cardio-oncology recommendations, GLS appears to be a sensitive marker of early systolic dysfunction.

Nevertheless, given the small sample size, the observed associations should be considered hypothesis-generating rather than definitive.

### Atrial strain as a complementary parameter

An important finding of this study is the independent association between LASr and cardiotoxicity. Reduced LASr values were associated with increased risk, suggesting early impairment of atrial reservoir function and ventricular compliance. This observation is consistent with emerging evidence that left atrial mechanics are sensitive to changes in left ventricular filling pressure and early diastolic dysfunction during anthracycline therapy.

The addition of LASr to LVGLS significantly improved model discrimination (AUC increased from 0.862 to 0.931), indicating that atrial mechanics may provide complementary pathophysiological information beyond ventricular deformation. However, given the limited number of events, routine clinical implementation cannot be recommended without further validation.

### Biochemical injury and troponin

High-sensitivity troponin I (hs-TnI) demonstrated the highest individual AUC among the parameters studied, consistent with prior evidence indicating that troponin elevation reflects early myocardial injury during anthracycline therapy [12, 13].

In nested ROC analysis, the addition of hs-TnI to the LVGLS + LASr model increased the apparent AUC to 0.979. However, the incremental improvement compared with the dual-parameter model reached only borderline statistical significance (P = 0.050). Furthermore, bootstrap internal validation demonstrated attenuation of model performance, suggesting the presence of optimism related to the limited sample size.

Thus, although the multimodal strategy appears promising, the incremental value of adding troponin to combined strain assessment should be interpreted with caution.

### Multimodal strategy: hypothesis-generating evidence

The stepwise improvement observed across the nested models suggests that myocardial deformation and biochemical injury markers capture distinct yet complementary aspects of anthracycline-related myocardial damage. Ventricular strain reflects mechanical dysfunction, atrial reservoir strain reflects ventricular filling pressures and compliance, and troponin levels reflect direct myocardial injury.

However, given the limited number of cardiotoxicity events (n = 15), these findings should be considered exploratory. The high apparent AUC values, particularly for the multimodal model, likely reflect partial optimism inherent to small datasets.

Internal validation using bootstrap resampling demonstrated attenuation in discrimination and reduced calibration stability, reinforcing the need for cautious interpretation. Despite relatively preserved discrimination after optimism correction, calibration analysis indicated deviation from the ideal calibration line, suggesting limited reliability of the model for individualized risk prediction.

### Clinical implications

If validated in larger prospective studies, this multimodal approach could have direct implications for clinical management by identifying a high-risk subgroup early after chemotherapy. Patients exhibiting a significant decline in LVGLS, elevated hs-TnI levels, and impaired LASr may benefit from closer surveillance and consideration of guideline-directed cardioprotective therapy (e.g., angiotensin-converting enzyme inhibitors or beta-blockers), even in the absence of overt LVEF decline.

This “treat the strain/troponin” concept is currently being explored in clinical trials, and our findings provide additional support for identifying patients who may benefit from early intervention.

Although definitive clinical recommendations cannot be derived from this pilot study, the results support further investigation of integrated strain- and biomarker-based surveillance strategies. If confirmed in larger prospective cohorts, such an approach may enable earlier risk stratification and more individualized cardioprotective management.

## Limitations

This study has several important limitations.

First, the relatively small sample size and limited number of outcome events (n = 15) reduce the statistical power and stability of the multivariable regression analysis, increasing the risk of model overfitting. Accordingly, the observed associations should be interpreted as preliminary and hypothesis-generating. Larger prospective studies with predefined power calculations are needed to validate these findings.

Second, although internal validation was performed using bootstrap resampling, no external validation cohort was included, limiting the generalizability of the results.

Third, cardiotoxicity was defined based on a single 1-month follow-up echocardiographic assessment. While this allowed evaluation of very early changes, it does not fully align with consensus definitions of cancer therapy-related cardiac dysfunction (CTRCD), which require confirmation on serial imaging. Therefore, our findings pertain specifically to early subclinical alterations, and their ability to predict sustained or late cardiotoxicity remains uncertain.

Fourth, follow-up was limited to the early post-chemotherapy period, and long-term cardiovascular outcomes were not assessed.

Fifth, left atrial strain was derived from standard left ventricular-focused apical views rather than dedicated left atrial views, which may have introduced measurement variability and potential underestimation of LA strain values.

Sixth, the initiation of cardioprotective therapy was not standardized and may have influenced subsequent cardiac function, representing a potential confounding factor.

Future multicenter prospective studies with larger sample sizes, adequate event rates, and external validation cohorts are required to confirm these findings.

## Conclusions

In this exploratory cohort of patients with breast cancer receiving anthracycline therapy, a multimodal strategy integrating LVGLS, LASr, and hs-TnI was associated with improved discrimination of early chemotherapy-induced cardiotoxicity compared with single-parameter assessment. These findings suggest that ventricular deformation, atrial mechanics, and biochemical markers provide complementary insights into early myocardial injury.

However, given the limited sample size and evidence of model optimism, these results should be interpreted with caution. Larger prospective studies with external validation are required before routine clinical implementation.

## Data Availability

No datasets were generated or analysed during the current study.

## Abbreviations

ACE: Angiotensin-converting enzyme
aOR: Adjusted odds ratio
AUC: Area under the curve
CI: Confidence interval
CTRCD: Cancer therapy-related cardiac dysfunction
ESC: European Society of Cardiology
GLS: Global longitudinal strain
hs-CRP: High-sensitivity C-reactive protein
hs-TnI: High-sensitivity troponin I
ICC: Intraclass correlation coefficient
IQR: Interquartile range
LA: Left atrial
LASr: Left atrial reservoir strain
LV: Left ventricular
LVEF: Left ventricular ejection fraction
LVGLS: Left ventricular global longitudinal strain
NT-proBNP: N-terminal pro–brain natriuretic peptide
ROC: Receiver operating characteristic
RVGLS: Right ventricular global longitudinal strain
TAPSE: Tricuspid annular plane systolic excursion
TTE: Transthoracic echocardiography
VIF: Variance inflation factor
